# Xerostomia and Salivary Flow in Patients Taking Antihypertensive Drugs

**DOI:** 10.3390/ijerph17072478

**Published:** 2020-04-05

**Authors:** Lucía Ramírez Martínez-Acitores, Fernando Hernández Ruiz de Azcárate, Elisabeth Casañas, Julia Serrano, Gonzalo Hernández, Rosa María López-Pintor

**Affiliations:** Department of Dental Clinical Specialities. ORALMED Research Group. School of Dentistry, Complutense University of Madrid, Plaza Ramón y Cajal s/n, 28040 Madrid, Spain; luciacitores@hotmail.com (L.R.M.-A.); fhruizac@hotmail.com (F.H.R.d.A.); ecasanasgil@gmail.com (E.C.); juliasv3@hotmail.com (J.S.); ghervall@ucm.es (G.H.)

**Keywords:** antihypertensives, hypertension, xerostomia, hyposalivation, salivary flow

## Abstract

The aims of this systematic review are (1) to compare the prevalence of xerostomia and hyposalivation between patients taking antihypertensive drugs with a control group (CG), (2) to compare salivary flow rate between patients treated with a CG, and (3) to identify which antihypertensives produce xerostomia. This systematic review was carried out according to the preferred reporting items for systematic reviews and meta-analyses (PRISMA) guidelines. To evaluate methodological quality of the eligible studies Cochrane Collaboration tool for assessing the risk of bias for clinical trials and the modified Newcastle–Ottawa scale case-control studies were used. The databases were searched for studies up to November 19th 2019. The search strategy yielded 6201 results and 13 publications were finally included (five clinical trials and eight case-control studies). The results of the included studies did not provide evidence to state that patients taking antihypertensives suffer more xerostomia or hyposalivation than patients not taking them. With regard to salivary flow, only two clinical studies showed a significant decrease in salivary flow and even one showed a significant increase after treatment. The case–control studies showed great variability in salivary flow, but in this case most studies showed how salivary flow is lower in patients medicated with antihypertensive drugs. The great variability of antihypertensive drugs included, the types of studies and the outcomes collected made it impossible to study which antihypertensive drug produces more salivary alterations. The quality assessment showed how each of the studies was of low methodological quality. Therefore, future studies about this topic are necessary to confirm whether antihypertensive drugs produce salivary alterations.

## 1. Introduction

Hypertension (HT) is a chronic medical condition in which blood pressure (BP) in the arteries is elevated. HT is currently defined as values in systolic BP > 140 mmHg and/or diastolic BP > 90 mmHg [1,2,3]. The prevalence of HT has increased substantially between 1990 and 2015 with a corresponding increase in deaths associated with this condition. In 2015, the Non-Communicable Diseases Risk Factor Collaboration (NCD RisC) estimated that 1.13 billion adults had HT [4]. Non-pharmacologic therapy with an appropriate lifestyle modification is recommended for all patients with HT. In addition, antihypertensive medication is recommended in many cases, and should be considered in others who have not achieved a goal BP despite non-pharmacologic therapy [5]. With regard to the current Guidelines for HT Management, among the first line antihypertensive drugs are angiotensin-converting enzyme (ACE) inhibitors, angiotensin receptor blockers, beta-blockers, calcium channel blockers, and diuretics [3].

Saliva is one of the most essential fluids of the body [6,7] with a number of important functions that are essential for maintaining oral health [8]. Xerostomia is a subjective complaint of dry mouth, whereas hyposalivation is an objective decrease of salivary flow [9]. There are a number of physiological situations that can alter salivary flow rate such as age, sex, body weight, number of teeth present in mouth, or time of day. Xerostomia takes place when taking certain drugs, radiotherapy treatment for head and neck cancer, chronic rheumatic diseases such as Sjögren’s syndrome, and other systemic disorders such as diabetes mellitus [7]. More than one thousand drugs are reported to be associated with xerostomia. Tricyclic antidepressants, muscarinic receptor antagonists, antipsychotics, opioids and benzodiazepines, antihypertensives, and antihistamines are the main medications producing this effect [10]. Among the antihypertensive drugs with a remarkable association with salivary alterations are β-adrenergic blockers, diuretics, ACE inhibitors, and drug combinations [1].

Although the side effects of antihypertensives have been widely studied [11,12,13], their effects on saliva have not been clarified. Therefore, the main objectives of this systematic review are: (1) to compare the prevalence of xerostomia and hyposalivation between patients taking antihypertensive drugs with a control group (CG), (2) to compare salivary flow rate between patients taking antihypertensive drugs with a CG, and (3) to identify which antihypertensives produce more xerostomia.

## 2. Materials and Methods 

The present systematic review was performed according to PRISMA (Preferred Reporting Items for Systematic Reviews and Meta-analyses) guidelines [14].

### 2.1. Focused Question

According to PRISMA guidelines, three focused questions were constructed. The questions were as follows: (1) Do patients taking antihypertensives have more xerostomia or hyposalivation than patients not taking them? (2) Is salivary flow lower in patients taking antihypertensives than in patients not taking them? (3) Do all antihypertensives reduce salivary flow in the same way?

### 2.2. Search Strategy

An exhaustive search of the literature was carried out, without prior limit restriction of the date until November 19th, 2019. Four international biomedical literature databases were used to perform the search: U.S National Library of Medicine (PubMed/MEDLINE), Web of Science (WOS), Cumulative Index to Nursing and Allied Health Literature (CINAHL), and the Cochrane Library. These databases were searched for studies using the following combination of terms: saliva, dry mouth, hyposalivation OR salivary flow AND hypertension, antihypertensive drug, OR antihypertensives. These terms were adjusted according to each database. An additional hand-search of the reference list of the reviewed articles was performed to find potential eligible studies.

**Figure 1:**
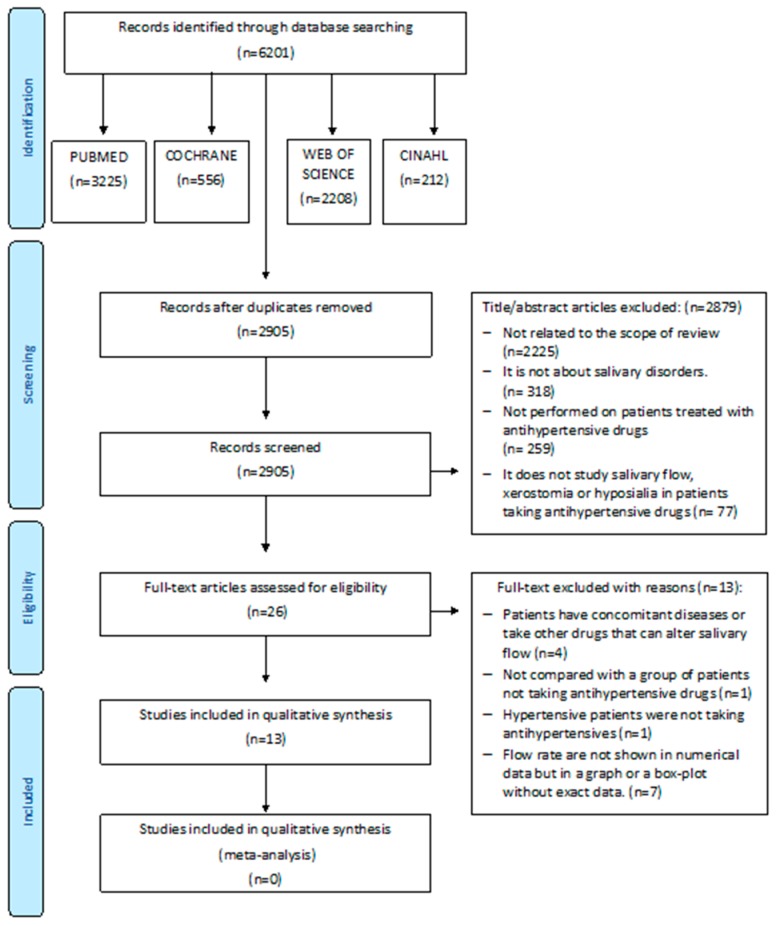
Flow chart.

Two independent researchers (LR and FH) compared search results to ensure completeness. They removed duplicated studies and screened full title and abstract of the remaining studies. A third reviewer (RMLP) resolved any differences in the selection of the studies (Figure 1).

### 2.3. Inclusion Criteria

Type of studies: studies had to be (1) original articles, (2) performed in humans, and (3) clinical trials, longitudinal and cross-sectional studies.

Types of population: We included studies conducted on patients who started antihypertensive therapy or were on antihypertensive therapy. Xerostomia, hyposalivation or salivary flow of these patients had to be compared with a control (CG) or placebo group that did not receive antihypertensives, did not suffer other diseases, and did not receive any other pharmacological treatments.

Outcomes: (1) Xerostomia or dry mouth was diagnosed based on different questions: Does your mouth usually feel dry? Does your mouth feel dry when eating a meal? Do you have difficulty swallowing dry food? Do you sip liquid to aid in swallowing dry food? Is the amount of saliva in your mouth too little most of the time? Does your mouth feel dry right now? Do you wake up at night to drink water? An affirmative response to one of these questions was enough to make a diagnosis of xerostomia. (2) Moreover, xerostomia could be quantified using the visual analogue scale (VAS) [15], xerostomia questions proposed by Fox et al. [16] or Xerostomia Inventory by Sreebney and Fox modified by De la Luz et al. [17]. (3) Hyposalivation was considered when stimulated whole saliva (SWS) was ≤0.7 mL/min or ≤0.5 mL/min. Unstimulated whole saliva (UWS) hyposalivation was considered when it was ≤0.3 mL/min, ≤0.2 mL/min, ≤0.15 mL/min, or when the modified Schimer test showed the strip color moved to 25 mm at 3 min. (4) Different types of salivary flow rates were considered by different saliva sampling: UWS, SWS, stimulated parotid saliva (SPS), unstimulated submandibular/sublingual saliva (USS), and stimulated submandibular/sublingual saliva (SSS). The amount of salivary flow could be expressed in mL/min or mg/min. 

### 2.4. Exclusion Criteria

Studies were excluded when (1) hypertensive patients were not taking antihypertensives; (2) studies were published in a language other than English and Spanish; (3) patients had concomitant diseases or took other drugs; (4) if the results were not compared with a placebo or CG of patients not taking antihypertensive drugs; and (5) if salivary flow rate was not shown in the numerical data but in a graph or a box-plot without the exact data.

### 2.5. Data Collection and Extraction

LR and FH independently extracted the data. Any differences in this phase were resolved by discussion with a third researcher (RMLP). The following data was collected: first author, publication year, country of origin, study population, mean age and gender, saliva sampling method, xerostomia and hyposalivation assessment, and type of antihypertensives. Outcomes extracted were the percentage of patients suffering xerostomia and/or hyposalivation, degree of xerostomia and salivary flow rate of patients taking antihypertensives, and placebo or CG. 

Statistical signification was given if available. For the clinical trials that presented intermediate assessments, we used the baseline scores and every follow-up score.

### 2.6. Risk of Bias in Individual Studies

Two independent researches (FH and LR) evaluated the methodological quality of each of the eligible studies using the Cochrane Collaboration tool for assessing risk of bias for clinical trials [14] and the modified Newcastle–Ottawa scale [18] for case-control studies.

The case-control studies and the clinical trials were classified in good, fair, or poor-quality following the score algorithm proposed by the Agency for Healthcare Research and Quality [19]. If there was disagreement between the two evaluators, a third reviewer (RMLP) was required.

### 2.7. Categorization of Studies

Due to the fact that two types of studies, clinical trials, and case-control studies were analyzed in this systematic review, the outcomes were grouped in two tables depending on the study design. 

### 2.8. Synthesis of the Results

Given the great heterogeneity of the results, it could not be possible to perform a meta-analysis. 

## 3. Results

### 3.1. Study Selection

The search strategy yielded 6201 studies (Figure 1). When the duplicates were removed, 2905 studies were left. Full title and abstract of each remaining article were screened individually. Twenty-six full-text articles were assessed for eligibility. Of these, 13 were discarded (see reasons in Figure 1). Finally, 13 publications were analyzed in full text.

### 3.2. Study Characteristics

Detailed information about the studies is shown in Table 1. Regarding the study design, five publications were clinical trials and eight case-control studies. The largest sample was collected by De la luz et al. [17] (*n* = 440) and the smallest by Nederfors et al. [20] (*n* = 12). Gender among these studies was heterogeneous; there were also publications where gender was not available [21,22,23]. 

Age of patients of the included studies was also heterogeneous. There were five studies that included people ≥ 60 years old [17,23,24,25,26], three studies in where people included was younger than 34 years old [20,27,28], three studies in where the age was between 46 and 54 years old [11,29,30], and one study in which the age ranged between 30 and 70 years old [22]. There was a publication where age of patients was not available [21].

The xerostomia percentage was analyzed in two studies [17,25]. The xerostomia level was analyzed only in three studies [17,20,25]. They used three different tools: VAS [20,25], xerostomia questions proposed by Fox et al. [25] and other questionnaires like Xerostomia Inventory by Sreebney and Fox modified by De la Luz et al. [17]. Hyposalivation was evaluated in four studies [11,17,22,25].

There were five studies in which UWS flow rate were collected [21,22,27,29,30], one study collected SWS flow rate [25], one SPS flow rate [24], four UWS and SWS [11,17,23,26], and two studies collected UWS, SWS, SPS, and SSS [20,28]. The UWS flow rate was the most collected, with 11 studies. 

Different methods for saliva collection were used: the spitting method [11,17,20,23,25,26,28,29,30], Carlson–Crittenden cups [20,24,28], Nederfors modified device [20,28], the cotton-wool method by Dollery et al. [27], a modified Schirmer test [25], and the Navazesh method [22]. One study did not show the method for saliva collection [21].

The schedule for saliva collection was before 12 a.m. in the majority of the studies, and five studies did not show the schedule for saliva collection [21,22,23,27].

Clinical trials collected the saliva at different times. Two studies evaluated salivary flow at one day, one week, and 4 weeks after treatment [29,30]. One study collected saliva 7 days after treatment [20]. One study evaluated the flow rate at 1 and 7 days after treatment [28]. Additionally, the last one evaluated the flow rate after administering an antihypertensive intravenous injection [27].

The type of antihypertensives studied in the reviewed publications included β-adrenergic blockers [22,25,27,29,30], diuretics [11,20,21,22,24,25], α-adrenergic blockers [22,27], calcium channel blockers [22,25], and heart glycosides [22]. In three studies, the type of antihypertensives was not available [17,23,26].

### 3.3. Risk of Bias within Studies

Regarding quality assessment, the clinical trials assessed presented many unclear biases, therefore we considered that they were poor quality studies. As we reflected in Table 2, the major problems were that the parameters included in the Cochrane risk assessment tool have not been described, so according to the tool the suitable score for these cases was “unclear”. 

On the other hand, quality assessment of five case-control studies obtained scores ranging from 4 to 5, so they were classified as poor quality [11,17,21,23,25]. Quality assessment of other three case-control studies obtained scores of 6, so they were classified as fair quality [22,24,26]. The main biases were present in the selection parameters (Table 3).

### 3.4. Main Findings

The results of the studies were divided in two groups depending on the type of study (clinical trial (Table 4) or case-control (Table 5)).

#### 3.4.1. Clinical Trials (Table 4)

The study performed by Nederfors et al. 2004 using bendroflumethiazide as an antihypertensive was the only study that analyzed the degree of xerostomia. They observed that patients treated with thiazide or furosemide increased xerostomia levels [20]. 

The majority of the studies evaluated UWS flow rate. Three studies showed a non-significant UWS increase after treatment with β-adrenergic blockers [29,30] or ACE inhibitors [28]. One study obtained a statistically significant decrease in UWS in normotensives treated with propranolol and phentolamine [27]. Additionally, the last study showed a no significant UWS decrease seven days after treatment with diuretics [20]. 

With regard to the SWS, there were only two studies. One of them presented a non-significant decrease in hypertensive patients treated with furosemide or bendroflumathiazide [20]. The other one obtained a non-significant increase after treatment with captopril [28]. 

Of the two studies that analyzed SPS flow rate, one obtained an SPS flow rate statistically elevated after treatment with captopril [28]. The other one did not obtain significant changes after treatment with bendroflumethiazide [20].

For the SSS the results were also heterogeneous, showing one study that did not have significant changes in patients treated with captopril [28] and the other one had a significant decrease after treatment with furosemide and bendroflumethiazide [20]. 

#### 3.4.2. Case-Control Studies (Table 5)

Xerostomia was studied in two articles [17,25]. In one of them [25] the percentage and degree of xerostomia were collected in patients treated with ACE inhibitors, calcium channel blockers, β-adrenergic blockers, and diuretics obtaining a statistically significant higher percentage and level of xerostomia in the hypertensive group. The other study only showed that the percentage of xerostomia was significantly greater in patients receiving antihypertensives, but it did not describe the drugs used [17].

Nonzee et al. 2012 was the only study that presented the percentage of patients with hyposalivation, being significantly greater in hypertensive patients treated with ACE inhibitors, calcium channel blockers, β-adrenergic blockers, and diuretics [25].

Six studies analyzed the UWS flow rate [11,17,21,22,23,26] and in all of them patients receiving antihypertensives had a lower salivary flow than CG, acquiring statistical significance in three of them [11,17,22].

Five publications studied SWS flow rate [11,17,23,25,26]. In four of them SWS [11,17,25,26] flow rate was lower in patients receiving antihypertensives, acquiring statistical signification in three of them [11,17,25].

SPS was recorded in one study and the hypertensive patients were treated with diuretics [24]. SPS flow rate was significantly lower in patients treated with hydrochlorothiazide. 

## 4. Discussion 

This systematic review showed the available evidence about a possible relationship between taking antihypertensives and xerostomia, hyposalivation, and a decrease in the salivary flow rate. Xerostomia and hyposalivation can have a detrimental effect on a patient’s quality of life leading to situations such as stress or anxiety [9]. Furthermore, a salivary flow decrease can increase susceptibility to dental caries or oral fungal infections, so these conditions must be given the importance they deserve [7].

The results of this study show that the possible relationship between antihypertensive intake and salivary alterations is not clear. The studies that deal with this topic were not abundant and in addition the existing ones did not have a high methodological quality. Besides, the available clinical trials about this topic were not current. The most recent was from 2006 [30] and the oldest one from 1981 [29]. In general terms, study design was heterogeneous from sample size, antihypertensive drugs used, duration of the study, type of salivary flow collection, or the saliva sampling method. Clinical trials included in this study had a small sample and the sample size was not calculated in any of them. In the case–control studies, the samples are higher in number, but the majority of the studies did not give a detailed explanation of the method used to obtain the sample.

According to the studies that evaluated hyposalivation [11,17,22,25], the UWS and SWS cut-off values are heterogeneous, and the majority not those currently used. Therefore, we believe that further investigation would be necessary about this topic.

Only three studies [17,20,25], included in this systematic review, assessed xerostomia. Currently, there are different xerostomia assessment questionnaires but the only one validated to measure xerostomia level in patients receiving drugs is the Xerostomia Inventory [31] and is probably the best for researching on medication-induced salivary gland disorders [9]. In this systematic review, none of the studies used this test to assess the level of xerostomia. However, De la Luz et al. [17] used a modified Xerostomia Inventory different from the one validated by Thomson et al. 

The types of saliva collected were different between the studies. Individual gland secretions are superior to whole saliva for many compositional analyses, because whole saliva contains non-salivary elements such as desquamated epithelial cells, food debris, bacteria, gingival crevicular fluid, and leukocytes. However, for the assessment of overall salivary gland dysfunction, whole saliva (UWS and SWS) is superior and clinically more relevant [32]. Furthermore, Navazesh stated in their study the methods for collecting saliva were that patients avoid smoking, eating or drinking 1–2 h before the appointment, and to remember that salivary flow rate is affected by a seasonal and diurnal factor [9,32]. For this reason, it is important to standardize the time of the day for saliva collection, and should be considered in long-term salivary studies about this topic. The schedule for saliva collection was before 12 am in the majority of the studies, except in five studies that did not show the time for saliva collection [21,22,23,27].

According to clinical trials, xerostomia was only studied in the Nederfors et al. study [20]. The level of xerostomia in the placebo group maintained a similar level while the two antihypertensive groups (patients treated with thiazide or furosemide) increased the xerostomia level. Hyposalivation was not studied in any. All the studies measured UWS flow rate; however only one showed a statistically significant UWS flow rate decreased in patients treated with an α-Adrenergic blocker and β-Adrenergic blocker [27]. Two studies measured SWS, SPS, and SSS flow rate [20,28]. One study showed a significant increase in SPS flow rate in patients treated with ACE inhibitors [28]. Additionally, the last one showed a significant SSS flow rate decrease in patients treated with diuretics [20].

For the case-control studies, the results are more logical regarding the current knowledge. Hyposalivation was studied in one manuscript, and it showed a greater number among patients taking antihypertensives than in CG [25]. Two articles studied xerostomia [17,25], and it was also higher in hypertensive patients than CG. Six studies measured UWS flow rate. Five of them showed a UWS decrease [11,17,21,22,23] acquiring statistical significance in three studies [11,17,22]. However, one of the studies showed a UWS flow increase [26]. Five studies measured the SWS flow rate. Three of them showed a significant SWS decrease [11,17,25]. One study measured SPS [24] obtaining a significant decrease in patients treated with diuretics.

The type of antihypertensives studied in case-control studies included β-adrenergic blockers, α-adrenergic blockers, diuretics, ACE inhibitors, calcium channel blockers, heart glycosides, and antihypertensive drugs with central effects. Due to the great heterogeneity of the study designs, and the different outcomes it was impossible to ensure if one antihypertensive produces more xerostomic effects than other.

Our review, thus far, had some limitations. Studies published in languages other than English and Spanish were not included. We also excluded the studies if the salivary flow rate was not shown in numerical data but in a graph or a box-plot without exact data. Another limitation is that we have also not been able to evaluate whether the duration of treatment influences the appearance of salivary disorders because case–control studies did not offer the time these patients had been on antihypertensive treatment. Finally, we did not include studies whose patients had concomitant diseases or took other drugs that can alter salivary flow. So, we could not evaluate if antihypertensives used in combination with other drugs could increase salivary disorders.

## 5. Conclusions

The available literature about this topic is scarce and based on the quality assessment performed we believe that there is a need for future research on this subject. Correct methodological studies with an adequate sample calculation to provide strong evidence should be performed. Long-term clinical trials are also needed to analyze if the effects of xerostomia/hyposalivation emerge after larger periods of time. It could be interesting to carry out long-term randomized clinical trials in which different antihypertensives at standardized doses were tested against a placebo/CG to elucidate which drug further reduces salivary flow. Future studies could help to understand which antihypertensive is more suitable for patients in terms of oral dryness, always within the limits of the drug´s indication.

Trying to respond to our focused questions we could say that: (1) With the current literature it was not possible to assure that patients taking antihypertensives have more xerostomia or hyposalivation than patients not taking antihypertensives. (2) With respect to the salivary flow rate, only two clinical trials found a statistically significant decrease in the flow rate after antihypertensive treatment, and one clinical trial showed a statistically significant increase in flow rate after antihypertensive treatment. So, we could not say that antihypertensives reduced salivary flow. The possible decrease could not be confirmed by case–control studies due to the great variability of saliva collection, but most studies found less salivary flow in the antihypertensive treatment group than in CG. (3) Finally, the type of antihypertensives studied in the reviewed publications included β-adrenergic blockers, α-adrenergic blockers, diuretics, ACE inhibitors, calcium channel blockers, heart glycosides, and antihypertensive drugs with central effects. Due to the great heterogeneity of the types of antihypertensives, the study design and the different outcomes made it impossible to ensure if one antihypertensive produced more xerostomic effect than other. Due to these results, future studies about this topic are necessary to confirm if antihypertensive drugs produce salivary alterations.

## Figures and Tables

**Table 1:** General characteristics of the studies.

Author, Year and Country	Study Design	Duration	Sample	Age and Gender	Type of Salivary Flow Rate	Saliva Sampling	Hyposalivation	Xerostomia Assessment	Type of Antihypertensive
Ben-Aryeh et al., 1981, Israel [29]	Clinical trial	6 weeks	10 CG 10 HT	12 female/8 male Mean age 54 years	UWS	8–9 am Spitting method 10 min	-	-	β-Adrenergic blocker (Pindolol)
Van Hoof et al., 1983, The Netherlands [27]	Clinical trial	1 day	23 CG 19 HT	42 Male 22–34 years	UWS	15 min cotton-wool method by. Dollery et al.	-	-	β-Adrenergic blocker (Propanolol) α-Adrenergic blocker (Phentolamine)
Streckfus et al., 1994, USA [24]	Case-control study	-	15 CG 20 HT	NT: 9 female/ 6 male Mean age 69.5 years HCTZ: 10 female/10 male Mean age 68.5 years	SPS	8–12 am Carlson–Crittenden cups: parotid	-	-	Diuretic (HCTZ)
Nederfors et al., 1995, Sweden [28]	Double blind, cross-over randomized trial	3 months	24 Healthy patients: Placebo Captopril	13 female/ 11 male Mean age 24 years	UWS SWS SPS SSS	7.30–8.30 am UWS: Spitting method 5 min SWS: Spitting method paraffin chewing 5min SPS: Modified Carlson–Crittenden cups SSS: Nederfors modified device 7.30–8.30 am	-	-	ACE inhibitors (Captopril)
Nederfors et al., 2004, Sweden [20]	Cross- over clinical trial	3 months	12 Healthy patients: Placebo Thiazide Furosemide	12 female Mean age 28 years	UWS SWS SPS SSS	UWS: Spitting method 5 min SWS: Spitting method paraffin chewing 5min SPS: Modified Carlson–Crittenden cups SSS: Nederfors modified device	-	VAS	Diuretic (Thiazide, furosemide)
Tahrir et al., 2006, Irak [30]	Clinical trial	4 weeks	48 HT treated with atenolol 48 CG	20 male /28 female Mean age 49 years	UWS	8–9 am Spitting method 10 min	-	-	β-Adrenergic blockers: Atenolol
Nonzee et al., 2012, Thailand [25]	Case-control study	-	200 HT 200 CG	CG: 118 female/ 82 male Mean age 58.82 ± 7.84 years HT: 104 female/96 male Mean age 62.41 ± 8.75 years	SWS	8–12 am UWS: Modified Schirmer test: 3 min SWS: Spitting method paraffin chewing 5min	Fontana et al. Hyposalivation SWS was diagnosed if the color of Schirmer text moved 25 mm at 3 min	Xerostomia questionnaire Fox et al. + VAS	β-Adrenergic blockers (Propanolol, atenolol) Diuretic (HCTZ) ACE inhibitors (Enalapril) Calcium channel blocker (Amlodipine)
Muñoz et al., 2012, Chile, [21]	Case-control study	-	14 HT 10 CG	Gender not available Age not available	UWS	Not available 1 min	-	-	Diuretics
De la luz et al., 2013, Mexico [17]	Case-control study	-	440 Patients: CG HT	268 female/ 172 male Mean age 68.34 ± 6.19 years	UWS SWS	Morning UWS: Spitting method 3 min SWS: Spitting method Chewing no available 5min	UWS < 0.15 mL/min SWS < 0.5 mL/min	Modified Sreebney and Fox questionnaire	Not available
Kagawa et al., 2013, Japan [26]	Case-control study	-	96 CG 9 Normotensive treated with antihypertensives drugs 18 HT	92 female/73 male Mean age 66.6 years	UWS SWS	10 am-3 pm UWS: Spitting method 5 min SWS: Spitting method paraffin chewing 2 min	-	-	Not available
Prasanthi et al., 2014, India [11]	Case-control study	-	50 CG 50 HT	CG 27 female/23 male Mean age 43.9 ±2.4 years HT 23 female/27 male Mean age 46.3 ±2.7 years	UWS SWS	9–10 am UWS: Spitting method 5 min SWS: Spitting method paraffin chewing 5 min	UWS<0.3 mL/min SWS<0.7 mL/min	-	Diuretics
Ivanovski et al., 2015, Republic of Macedonia [22]	Case-control study	-	30 CG 30 HT	Gender not available 30–70 years	UWS	Navazesh method 10 min.	USW<0.2 mL/min	-	Diuretic β-Adrenergic blockers α-Adrenergic blocker ACE inhibitors Calcium channel blocker Heart glycosides Antihypertensives drugs with central effect
Nimma et al. 2016, India [23]	Case-control study	-	20 CG 20 HT	Gender not available 60–75 years	UWS SWS	Moment no available UWS: Spitting method 5 min SWS: Spitting method paraffin chewing 5 min	-	-	Not available

UWS (unstimulated whole saliva), SWS (stimulated whole saliva), SPS (stimulated parotid saliva), USS (unstimulated submandibular/sublingual saliva), SSS (stimulated submandibular/sublingual saliva), HT (hypertension treated with antihypertensives), GC (control group), DM (diabetes mellitus), HCTZ (hydrochlorothiazide), BD (blood pressure).

**Table 2:** The Cochrane risk of bias of the included clinical trials.

	Random Sequence Generation	Allocation Concealment	Blinding of Participants and Personnel	Blinding of Outcome Assessment	Incomplete Outcome Data	Selective Reporting	Other Bias	Quality
**Ben-Aryeh 1981**	High	High	High	High	Unclear	Unclear	Unclear	Poor quality
**Van Hoof et al. 1983**	Unclear	Unclear	High	Unclear	Unclear	Unclear	Unclear	Poor quality
**Nederfors et al. 1995**	Unclear	Unclear	Low	Unclear	Unclear	Low	Unclear	Poor quality
**Nederfors et al. 2004**	Unclear	Unclear	Low	Unclear	Unclear	Low	Unclear	Poor quality
**Tahir et al. 2006**	High	High	High	High	Unclear	Unclear	Unclear	Poor quality

**Table 3:** The modified Newcastle–Ottawa scale for case-control studies.

	Selection (1)	Selection (2)	Selection (3)	Selection (4)	Comparability (1)	Outcome (1)	Outcome (2)	Score	Quality
Streckfus et al. 1994b [24]	*	-	-	*	*	**	*	6/10	Fair
Nonzee et al. 2012 [25]	*	-	-	-	*	**	*	5/10	Poor
Muñoz et al. 2012 [21]	*	-	-	-	*	*	*	4/10	Poor
De la luz et al. 2013 [17]	-	-	-	-	*	**	*	4/10	Poor
Kagawa et al. 2013 [26]	*	-	-	*	*	**	*	6/10	Fair
Prasanthi et al. 2014 [11]	*	-	-	-	*	*	*	4/10	Poor
Ivanovski et al 2015 [22]	*	-	-	*	*	**	*	6/10	Fair
Nimma et al. 2016 [23]	-	-	-	*	*	*	*	4/10	Poor

* *p* < 0.001; ** *p* ≤ 0.05.

**Table 4:** Clinical trials results.

Author/Year/Country	Antihypertensive Medications	Salivary Flow Rate (mL/min) (g/min)									
		Experimental					No Treatment	Placebo			Results
		Before		After				Before	After		
Ben-Aryeh et al., 1981, Israel [29]	β-Adrenergic blocker (Pindolol)	UWS: 0.24 ± 0.14		3 h	24 h	6 weeks	UWS: 0.39 ± 0.18				The UWS flow rate increased, no significantly, in HT patients treated with pinilol. However CG salivary flow were higher than experimental group.
				0.31 ± 0.11	0.27 ± 0.2	0.36 ± 0.15					
Van Hoof et al. 1983 The Netherlands [27]	Intravenous injection of propranolol			1 mg		*5 mg*	NT: USW: 0.85 ± 0.08 * BHT: USW: 0.51 ± 0.04 *				UWS flow rate significantly decreased in NT patients treatment with propranolol and phentolamine. Salivary UWS flow rate was significantly lower in BHT patients no treatment than normotensive patients.
		NT: UWS: 0.88 ± 0.15		0.72 ± 0.12**		*0.77* ± 0.12					
		BHT: UWS: 0.34 ± 0.04		0.32 ± 0.05		*0.34* ± 0.04					
	Intravenous injection of phentolamine			1 mg		5 mg					
		NT: UWS: 0.88 ± 0.15		0.74 ± 0.14**		0.77 ± 0.11					
		BHT: UWS: 0.54 ± 0.14		0.47 ± 0.10		0.53 ± 0.10					
Nederfors et al. 1995 Sweden [28]	ACE inhibitors (Captopril)	UWS:0.59 ± 0.24 SWS:1.67 ± 0.57 SPS:1.41 ± 0.77 SSS:1.39 ± 0.51		*Day 1*		*Day 7*	-	UWS: 0.64 ± 0.57 SWS: 1.84 ± 0.60 SPS: 1.61 ± 0.82 SSS: 1.35 ± 0.63	*Day 1*	*Day 7*	SPS is significantly higher in patients treated with captopril
				UWS: 0.65 ± 0.27 SWS: 1.79 ± 0.47 SSP: 1.44 ± 0.84 ** SSS: 1.38 ± 0.71		UWS: 0.69 ± 0.69 SWS: 1.85 ± 0.46 SSP: 1.86 ± 0.91** SSS: 1.41 ± 0.62			UWS: 0.65 ± 0.29 SWS: 1.95 ± 0.72 SPS: 1.62 ± 0.70 SSS: 1.57 ± 0.64	UWS: 0.62 ± 0.28 SWS: 1.81 ± 0.68 SPS: 1.56 ± 0.87 SSS: 1.57 ± 0.74	
Nederfors et al. 2004 Sweden [20]	Diuretic (Thiazide, furosemide)			Day 7					Day 7		SSS was significantly affected, statistically (P < 0.05) decreased in the morning during chronic treatment with both drugs. The percentage reduction in SSS was 26 and 24% for bendroflumethiazide and furosemide, respectively.
		Furosemide UWS: 0.31 ± 0.12 SWS: 1.37 ± 0.54 SPS: 0.81 ± 0.44 SSS: 1.41 ± 0.57	Bendroflumethiazide UWS: 0.34 ± 0.14 SWS: 1.34 ± 0.39 SPS: 0.76 ± 0.44 SSS: 1.27 ± 0.54	Furosemide UWS: 0.29 ± 0.09 SWS: 1.34 ± 0.42 SPS: 0.83 ± 0.53 SSS: 1.05 ± 0.46**		Bendroflumethiazide UWS: 0.30 ± 0.14 SWS: 1.29 ± 0.49 SPS: 0.76 ± 0.37 SSS: 0.96 ± 0.57**		UWS: 0.31 ± 0.12 SWS: 1.40 ± 0.39 SPS: 0.67 ± 0.12 SSS: 1.18 ± 0.56	UWS: 0.37 ± 0.20 SWS: 1.36 ± 0.37 SPS: 0.75 ± 0.42 SSS: 1.12 ± 0.49		
Tahrir et al. 2006 Irak [30]	β-Adrenergic blockers: Atenolol	UWS 0.24 ± 0.14		24 h	1 week	4 weeks	UWS: 0.38 ± 0.18				The UWS flow rate increased, not significantly, in HT patients treated with atenolol
				0.26 ± 0.11	0.28 ± 0.2	0.33 ± 0.15					

* *p* < 0.001; **p ≤ 0.05; UWS (unstimulated whole saliva), SWS (stimulated whole saliva), SPS (stimulated parotid saliva), USS (unstimulated submandibular/sublingual saliva), SSS (stimulated submandibular/sublingual saliva), NT (normotensive), BHT (borderline hypertensive).

**Table 5:** Case-control studies results.

Author/Year	Antihypertensive Medications	UWS (mL/min)	SWS (mL/min)	SPS (mL/min)	Hyposalivation Salivary Flow Rate	Hyposalivation (%)	Xerostomia (%)	Level of Xerostomia (cm)	*p* Value
Streckfus et al., 1994b, USA [24]	Diuretic (HCTZ)	-	-	CG: 0.695 ± 0.44 HT: 0.685 ± 0.39 HCTZ: 0.422 ± 0.24	Not available	-	-	-	0.02
Nonzee et al., 2012, Thailand [25]	β-Adrenergic blockers (Propanolol, atenolol) Diuretic (HCTZ) ACE inhibitor (Enalapril) Calcium channel blocker (Amlodipine)	-	CG: 1.31 ± 0.34 HT: 0.73 ± 0.30	-	SWS hyposalivation was diagnosed if the color moved 25 mm at 3 min according to Fontana et al.	CG: 5% HT: 57%	CG: 25.5% HT: 50%	CG: 1.53 ± 1.89 HT: 3.32 ± 2.72	0.05
Muñoz et al., 2012, Chile [21]	Diuretics	CG: 1.92 ± 0.40 HT: 0.57 ± 0.29	-	-	Not available	-	-	-	0.13
De la luz et al., 2013, Mexico [17]	Not available	CG: 0.31 ± 0.17 HT: 0.27 ± 0.17	CG: 1.33 ± 0.70 HT: 1.12 ± 0.62	-	UWS < 0.15 mL/min SWS < 0.5 mL/min	-	CG: 12.7% HT: 23.6%		UWS: 0.023 SWS: 0.001 Xerostomia 0.001
Kagawa et al., 2013, Japan [26]	Not available	CG: 0.32 (0.19–0.51) HT: 0.35 (0.23–0.57)	CG: 1.66 (1.18–2.39) HT: 1.53 (1.01–2.07)	-	Not available	-	-	-	UWS: 0.85 SSS: 0.39
Prasanthi et al., 2014 India [11]	Diuretics	CG: 2.16 ± 0.72 HT: 0.88 ± 0.41	CG: 7.90 ± 1.87 HT: 2.71 ± 1.08	-	UWS < 0.3 mL/min SWS < 0.7 mL/min	-	-	-	0.001
Ivanovski et al., 2015, Republic of Macedonia, [22]	Diuretics β-Adrenergic blockers α-Adrenergic blocker Angiotensin converting enzyme inhibitors Calcium channel blocker Heart glycosides Antihypertensives drugs with central effect	CG: 0.6 ± 0.1 HT: 0.3 ± 0.2	-	-	USW < 0.2 mL/min	-	-	-	0.000
Nimma et al., 2016, India [23]	Not available	CG: 2.73 ± 0.68 HT: 2.58 ± 0.37	CG: 3.30 ± 0.70 HT: 3.63 ± 0.65	-		-	-	-	UWS: 0.13 SWS: 0.39

HT: hypertensive patients; CG: normotensive patients; METRO: metropolol; ENA: enalapril; HCTZ: hydrochlorothiazide.
